# Reprogramming of Human Limbal Niche Progenitors

**DOI:** 10.7150/ijms.135665

**Published:** 2026-09-24

**Authors:** Xinghan Guo, Wei Wang, Allison Wilde, Yingting Zhu, Guigang Li

**Affiliations:** 1 Department of Ophthalmology, Tongji Hospital, Tongji Medical College, Huazhong University of Science and Technology, Wuhan 430030, China.; 2 Hubei Key Laboratory of Otolaryngologic and Ophthalmic Diseases, Wuhan, 430000, China.; 3 BioTissue, Miami, FL, 33126 USA.

**Keywords:** limbus, niche, stem cell, differentiation

## Abstract

Limbal niche cells (LNCs) possess embryonic stem cell (ESC) characteristics, expressing ESC markers such as Sox2, Nanog, and Oct4; stromal characteristics, expressing neurocrest markers such as CD90; and have angiogenic potential expressing angiogenesis markers such as CD34. In addition, these cells can be induced into adipocytes, chondrocytes, and osteocytes, indicating that they are valuable adult stem cells. However, how these cells may be appropriately passaged and maintained, what signaling pathways are necessary for such regeneration, and how the cells can be used for the treatment of various diseases caused by stem cell deficiency, such as limbal stem cell deficiency, remain unclear. Herein, we summarize the most recent advances in limbal niche research, with emphasis on the regeneration of adult limbal progenitors and their mechanisms, to promote cell-based therapy for various eye diseases due to stem cell deficiency or malfunction. Such a strategy may also provide a platform for stem cell therapy for other similar human diseases worldwide.

## Human Limbal Niche Cells

Limbal niche cells (LNCs) contain pigment cells (melanocytes) 1-3, neural crest cells, and vascular endothelial cells 4. Although LNCs were suggested in those studies, their presence was later discovered and confirmed by our studies using collagenase 5, 6. This enzymatic digestion technique resulted in the identification of LNCs as specific Vimentin (Vim)+ mesenchymal cells 5, 7. Specifically, we reported that dispase digestion alone removes the sheets composed of limbal epithelia 8 but was unable to take away all limbal epithelial progenitors. In contrast, collagenase A digestion can remove all limbal progenitors and underlying pancytokeratin (PCK)-, p63α-, Vim+ stromal cells, which are small in size and express ESC markers such as Sox2 and CD34. Intriguingly, Bhartiya *et al*. also reported highly potent stem cells, small in size, expressing embryonic stem cell markers such as Nanog and Sox-2 9. In addition, human Limbal Niche Cells (hLNCs) have angiogenic potential, expressing angiogenesis markers, Fetal Liver Kinase-1 (Flk-1), CD34, CD31, and PDGFRβ 10.

The vascular niche contains basement membrane (BM), cellular components such as vascular endothelial cells (VECs) and pericytes 11. The vascular BM contains laminin, collagen IV, and proteoglycans, with growth factors such as basic fibroblast growth factor (bFGF), epidermal growth factor (EGF), insulin-like growth factor (IGF), platelet-derived growth factor (PDGF), and transforming growth factor-β (TGF-β). Human SCs change from an osteoblastic to a vascular status to promote their replication and differentiation in the vascular BM 12, 13. Human ESCs may remain undifferentiated when cultivated on top of Matrigel 14, 15. BM promotes replication as well as differentiation in human stromal SCs *in vitro* 16, 17. VECs can be supportive of bone marrow SCs 18, the central nervous system 19, 20, muscles 21, and pancreatic islets 22. Their mechanisms involve physical contact and growth factors such as bFGF, Ang1, Bone Morphogenetic Protein 4 (BMP4), Desert Hedgehog Protein (DHH), Pigment Epithelium-Derived Factor (PEDF), NFC Data Exchange Format (NDEF), FGF4, and Stromal Cell-Derived Factor-1 (SDF-1) 12, 19, 20, 23. Nerve growth factor (NGF) signaling may also promote the proliferation of limbal progenitors, increase the efficiency of colony formation, and maintain the stem cell phenotype 24.

## MSCs within their Stem Cell Niche

Mesenchymal stem cells (MSCs) are located around perivascular areas of a number of tissues such as the bone marrow and the brain and spinal cord 25, the dental pulp 26, and skeletal muscle, pancreas, adipose tissue, and placenta 27. Although the cell origin of MSCs is not very clear, accumulating evidence suggests that the progenitor cells are located in perivascular sites 28-30. Interestingly, significantly different cell phenotypes are observed depending on cell location. For example, basal niche cells, in contrast to the cells around the rim, show distinct characteristics, suggesting that the fate of stem cells varies significantly depending on their position within or near the niche 31, likely due to differences in the microenvironment. Such a niche environment with more progenitors may be associated with the success rate of *in vitro* transplantation of epithelial stem cells because a higher number of autologous donor cells is closely associated with a higher transplantation success rate in the long term 32, and transplantation of limbal epithelial progenitors along with stromal cells is recommended for eye tissue repair and healing 33. Currently, the prevailing approaches for restoring the microenvironment of the ocular surface are (1) cell-based therapies using progenitors capable of supporting the limbal epithelial progenitors and modulating inflammation by MSCs, (2) regeneration of the microenvironment using a bioactive matrix from decellularized tissues, such as amniotic membrane (AM), or synthetic molecules, and (3) promotion of certain signaling pathways using cytokines and growth factors 34-37.

## HC-HA/PTX3 Complex

Human amniotic membrane (AM) may be applied at injury and surgical sites to inhibit inflammation, scarring (for reviews, see 38-40), and regulate the niche of limbal epithelial progenitors 41-44. Human AM may result in rapid regression of edema and inflammation of the corneal stroma 45-47. Subsequently, our studies demonstrated that AM extract and HC-HA, where HC refers to the heavy chain from inter-α-inhibitor and HA refers to hyaluronan. Such HC-HA extracted from AM has functions against inflammation and scarring 48, 49. Notably, HC-HA is more powerful against angiogenesis than HA by reducing migration, proliferation, differentiation, and viability without affecting Human Umbilical Vein Endothelial Cells (HUVECs) in culture 50. We have shown that HC-HA can be reconstituted *in vitro* using purified components, e.g., HA, IαI, TNF-stimulated gene-6 (TSG-6), and TNF-stimulated gene 14 (TSG-14) (also known as Pentraxin 3 [PTX3]) 51. For this purpose, others have added TSG-6 to create recombinant HC-HA (rcHC-HA) 52. We, however, find that recombinant HC-HA thus created is different from that created when PTX3 is added first to immobilized HA. TSG-6, an important mediator of anti-inflammation and tissue remodeling 53, 54, is not detectable but can be quickly induced by IL-1 and TNF-α in many cell types 54-56.

### HC-HA/PTX3 Supports Progenitor Status of Limbal Niche Cells

We have established a new approach for isolating and maintaining limbal epithelial progenitors and niche cells by digesting human limbus using collagenase 5. Such LNCs, accompanied by limbal epithelial progenitors (LEPs), exhibit characteristics of being vimentin-positive, pancytokeratin-negative, and expressing SC markers, for example, Nanog, CD34, N-cadherin, Rex1, and SSEA4 5, 57. Such cells might be passaged on top of Matrigel, in a stem cell culture medium, to as many as 12 passages, exhibiting characteristics of MSCs and angiogenic potential 10. When reseeded in three-dimensional (3D) cell culture matrix (Matrigel), these cells may revert to embryonic SC (ESC)-like cells. Based on the findings, we have established a 3D Matrigel model system *in vitro*, in which limbal epithelial progenitors (LEPs) may reunite with LNCs, resulting in sphere formation through activation of the SDF-1/CXCR4 (chemokine receptor type 4) cascade 7. When LNCs are in constant contact with LEPs, better clonal growth is observed when cultured on top of 3T3 layers 5, which prevents LEPs from differentiating into corneal endothelial cells 57, 58. Our studies demonstrated that activation of BMP and Wnt signaling pathways in both LNCs and LEPs, and also between LEPs and LNCs, resulted in LEPs' growth in the form of colonies. As a result, the spheres formed on the Matrigel inhibited corneal endothelial differentiation and promoted LEPs growth as colonies through the activation of the canonical Wnt pathway 59. With HC-HA/PTX3 immobilized on the culture plates, sphere formation also suppressed corneal endothelial differentiation, promoted epithelial quiescence, and inhibited LEPs growth in colonies 60. The results were linked to the inhibition of canonical Wnt and the activation of BMP and noncanonical Wnt pathways in both LNCs and LEPs. The BMP pathway from LNCs plays a critical role in the process, because inhibition of the BMP pathway in LEPs promotes cell cycle progression, inhibits self-renewal through Bmi-1, and increased LEPs growth in colonies. Therefore, HC-HA/PTX3 may activate the BMP pathway in both LNCs and LEPs, causing quiescence *in vivo* 61, 62 and *ex vivo* 63 while regulating the proliferation of LEPs, which might be a reliable candidate for the potential treatment of corneal blindness, for example, blindness caused by limbal stem cell deficiency (LSCD) 44, 64-66.

### BMP-MET Initiates Adult Progenitor Reprogramming

Mesenchymal to epithelial transition (MET) occurs when tissues are being built or remodeled during embryogenesis, tissue repair, tissue restoration, fibrogenesis, and neoplasia. Unfortunately, it is still largely unknown how MET occurs. Recently, Takahashi was the first to show that somatic cells may be reprogrammed through Sox2, Krüppel-like Factor 4 (Klf4), Oct4, and MYC to induced pluripotent stem cells (iPSCs) 67. This process of iPSC generation from adult mouse fibroblasts involves a change of cell shape from a spindle shape to tightly packed round clusters, resembling MET 67. In the initial phase of reprogramming, it is reported that upregulation of E-cadherin and occludin 68-70 and downregulation of snail, a MET regulator 71, play a crucial role in achieving close cell-cell contact 70. Ablation of endogenous E-cadherin reduces the reprogramming colony efficiency 68. *In vivo*, knockout of E-cadherin leads to cellular decompaction during the mouse preimplantation process in early embryogenesis 72. In our case, we did not detect any change in E-cadherin at the mRNA level in LNCs cultured on immobilized HC-HA/PTX3, indicating that factors other than E-cadherin are important during compaction. A previous report suggested that adult cellular reprogramming to iPSCs involves a mechanism in which reprogramming factors such as c-Myc, Oct4, and Sox2 inhibit Wnt and canonical TGF-β signaling 68. Because Zeb siRNA combined with Y-27632, a small molecule that attenuates Rho-associated protein kinase (ROCK) proteins (ROCK1 and ROCK2), can completely reverse epithelial-to-mesenchymal transition (EMT) through eliminating contractile actomyosin bundles, reducing stromal mRNA and protein expression, and restoring E-cadherin and Ksp-cadherin that maintain healthy tubular epithelial cells 73, and Y27632 inhibits proliferation and EMT as we reported recently 74, 75, we speculate that inhibition of Rho-ROCK signaling is also important for MET, quiescence, and cell shape change. In addition, we have recently summarized how to avoid EMT for generating functional human corneal endothelia from their progenitors 76 and determined that progenitors are present in the trabecular meshwork 77.

Takahashi and Yamanaka *et al*. have characterized that reprogramming of adult cells into iPSCs may be achieved by the Sox2, Klf4, Oct4, and c-Myc (SKOM) method 67. Three phases are involved in such reprogramming, i.e., the initiation, maturation, and stabilization phases (reviewed in 78). The initial phase of reprogramming involves cell shape changes from a spindle shape to tightly packed round clusters, resembling MET 67. In this phase, BMPs induce MET, causing significant shape changes; for example, the shape of fibroblasts may resemble that of epithelial cells 79, 80. Blockade of BMPs impairs the initiation phase of the reprogramming process 70, 80. The hallmark of MET change requires the downregulation of EMT regulators, such as snail1/2, slug, and Zeb1/2 81, and the upregulation of E-cadherin, occludin, Cdh1, Crb3, EpCAM, and the epithelial-associated miRNA-200 family 68, 70, 82. A recent study revealed that the miR-200 family promotes MET by suppressing Zeb2 and snail2. During these initial stages, several groups have demonstrated that cells acquire ESC-like proliferation, resistance to apoptosis, and changes in senescence cascade pathways 81, 83-85. For instance, the BMP-SMAD-ID pathway inhibits cell senescence by inhibiting p16/INK4A 80. In addition, a time-course study also disclosed that multiple markers, for instance, Thy1 (CD90), CD44, and ICAM1 (CD54), were lost while alkaline phosphatase and Tra-1-60 were gained. The epigenetic regulators Tet1, Tet2, and Tet3 are also required to activate the miR-200 family. Although the transition from the initiation phase to the next phase is not clearly defined, cells generally differentiate until the pluripotent genes are expressed. At this maturation phase, the detection of pluripotency-associated genes, including Fbxo15, Sall4, Oct4, and Nanog 70, can be used as a reliable assessment of successful reprogramming. It is suggested that adult cells may acquire pluripotent markers in a sequential way, in which markers such as Sox2 and CD31 are expressed during the stabilization phase. BMP signaling present in serum and feeders decreases iPSC generation and therefore must be downregulated or eliminated at the stabilization stage 80, and interestingly, Wnt activation is necessary in the late-stage reprogramming 86. In the stabilization phase, cells are self-renewed, endogenous pluripotent genes are reactivated, the inactive X chromosome is restarted, and telomeres are lengthened, which restore normal cellular and tissue functions 79. It remains unclear whether continuous BMP signaling during the stabilization phase promotes proper lineage differentiation (lineage-specific regeneration).

Studies have demonstrated that SCs in the niche compartments are regulated by several pathways, including BMP, Wnt, and Notch 87-90. Among those signaling pathways, BMP signaling can be blocked by noggin 90-92, gremlin1 (GREM1), GREM, and chordin-like 1 (CHRDL1) 93, 94. It is suggested that BMP signaling is closely mediated through BMP inhibitors, which prohibit BMPs from interacting with their receptors. These inhibitors may be classified into 3 main groups: (1) The BMP pro-region might fold back onto BMP proteins, act like a shield, thus blocking BMP interactions with their receptors; (2) Ligand inhibitors may bind to BMPs directly to form inactive complexes; and (3) Receptor inhibitors may interfere with BMP interactions with their receptors 95. Chemicals such as sclerostin, follistatin, and noggin have been used as BMP inhibitors for BMP mechanistic studies 96-100. Chordin and Connective Tissue Growth Factor (CTGF) are BMP antagonists that may act in the skeleton to regulate BMP availability 101. In addition, other BMP antagonists, for example, Cerberus has a head-inducing ability in *Xenopus* but is not crucial for knockout mice 102, 103; USAG-1 is a main regulatory protein that mediates tooth development and growth in a mouse model 104; Coco is responsible for left-right symmetry 105; and Prdc, Twisted, and Crossveinless-2 are associated with abnormal phenotypes, skeletal complications, and kidney and eye development 106. Furthermore, BMP receptor antagonists include BMP-3, which is produced by osteoblasts, acts within bone tissue, and inhibits BMP activation via the type II activin receptor, thereby retarding bone growth 107, 108.

### Cytoskeleton Changes Mediated by Rho GTPases

Compelling evidence suggests that both actin cytoskeletons and microtubules can be directly regulated by the Rho GTPase family (Rac, RhoA, Cdc42). RhoA can target ROCK (Rho kinase), which facilitates several downstream pathways involving cytoskeletal changes. First, ROCK can directly regulate the phosphorylation/dephosphorylation of the regulatory light chain (RLC) through a calcium-activated enzyme called myosin light chain kinase (MLCK) or the regulatory subunit of myosin phosphatase (MYPT1), respectively. This event prompts the activation of non-muscle myosin IIA and myosin IIB, which drive contractility of the cell cortex, cell motility, and disassembly of the actin network effectively at the cellular level. Another ROCK target is activated via LIM kinase (LIMK), which phosphorylates and inhibits cofilin. Activation of LIMK is not uniquely controlled by ROCK; however, downstream of activation of Rac1 and PAK can also activate LIMK or Testis-Associated Actin Remodeling Kinase 2 (TESK2), through phosphorylation at Ser3, thus inactivating the actin depolymerization factor (ADF)/cofilin. Meanwhile, the Slingshot (SSH) phosphatase and the HAD-family phosphatase, Chronophin (CIN), are dedicated to dephosphorylating cofilin. Interestingly, counteractions between Rac1 and RhoA may lead to different cellular behaviors (cellular heterogeneity), that is, they may act on identical genes but behave quite differently from one another, as demonstrated during epidermal morphogenesis. This study revealed that Rac1 through β-PIX/PIX-1 and PAK1, and RhoA, via ROCK/LET-502, may coordinate with each other to regulate apical junctions and the formation of basolateral protrusions during epidermal morphogenesis. Cell shape changes may be accomplished by generating physical forces through the following reactions: First, F-actin and myosin II work together to produce a pulling force with another cellular component, the cell cortex, in which E-cadherin acts as a linker between the inner network and the outside environment 109. F-actin, cellular fibers acting as an internal framework, work together with myosin, a cellular motor protein, to create cellular force and movement, with epithelial cells as an important linker of various organs such as skin 110-116. Rho GTPases turn on cellular switches by binding to GTP, becoming active, traveling to the plasma membrane, and triggering downstream cascades to mediate the cytoskeleton 117. Previous work has identified Guanine Nucleotide Exchange Factors (GEFs) as the activators of RhoA during development 112, 118, 119. For example, RhoA GEF2 proteins activate the RhoA pathway, which induces cellular shape changes with tissue folding during fruit fly embryonic development 118, 120. Apical constrictions push tall cells into a wedge-like state, that is, a flat layer becomes bent and folded 121, 122. Transcription factors, such as Snail and Twist, may promote G protein-coupled receptor signaling to mediate apical accumulation of RhoGEF2 120, 123-125. Apical RhoGEF2 acts as a switch to squeeze the apex of a cell, causing cell shape change. Myosin acts as a muscle within cells to lead to such a cellular change 113-116. Myosin also acts as a small motor to propel cells to change shape and move by repeated short pulses during terminal contractions 126. ROCK is also involved in cell shape change and movement in repeated beats 115, 116. Cellular pulses probably occur due to myosin motors turning on and off in cycles, during which ROCK activates myosin by mediating its phosphorylation and dephosphorylation 115, 116, and possibly F-actin assembly is also involved in the process 113. Myosin may cause F-actin contraction that dictates cellular shape changes 113, 126 and might help mediate cell signals in a timely manner at the correct cellular locations 116, 127. In addition, myosin activities alter over time, and RhoA helps interacting proteins to form certain patterns to mediate such a cascade 113. During this process, radial cell polarity (RCP) occurs, in which ROCK is rearranged in a unique pattern in the apical cellular compartment but absent at adherent junctions 113. RCP is dependent on Twist. Interestingly, RhoA stimulation is needed but sufficient by itself to promote cellular apical contraction and interaction of ROCK and myosin. RhoGEF2 triggering might occur ahead of myosin activation, indicating that RhoA plays a significant role in contractile pulses. Such apical constriction requires Cumberland GAP (C-GAP) to control the location and the timing of RhoA proteins' activation within cells 128. In fact, C-GAP controls whether the cell shape change is transient or stable by acting as a one-way lock 128, 129. In all, present evidence suggests that activation of RhoA alone cannot induce cellular constriction. In contrast, RhoA signaling regulated by its helper proteins, RhoGEF2 and C-GAP, is the main mechanism mediating apical contraction and tissue status, as well as mediating the GEF-to-GAP ratio for a one-way-lock constriction into correct tissue alignment 128, 129. Rac and Rho GTPases can control cell motility by changes in GTP hydrolytic activities, which are involved in cytoskeleton rearrangements 130.

### SDF-1/CXCR4 Signaling

In the stem cell-based repair, the SDF-1/CXCR4 pathway plays a significant role in guiding stem cells into wounded tissues across multiple organs 131-133. Late SDF-1 expression could contribute significantly to a therapeutic approach for tissue regeneration 131, 134. Acute injury may trigger SDF-1 release, which attracts natural tissue stem cells to the damaged sites for repair 135 and maintains target organ cells in a healthy state, for instance, protecting heart muscle cells 136. In 2011, Xie *et al*. from our lab reported that after cell-cell junctions were dissociated with trypsin and EDTA, individual NC and SC could be reassembled, leading to sphere formation on 3D Matrigel with embryonic SC culture medium, and that the expansion of spheres was mediated via SDF-1 produced by limbal epithelial progenitors and CXCR4 produced by limbal mesenchymal progenitors 7. CXCR4 attenuation via a CXCR4-neutralizing antibody or the CXCR4 inhibitor AMD3100 attenuated the reunion and produced small, separate spheres. The resultant epithelial spheres were significantly differentiated, with a loss of holoclones, indicating that NC association regulated via SDF-1 and CXCR4 is essential for limbal SC performance 7. We have recently shown that HC-HA/PTX3 may rejuvenate aging eye cells through important eye NC self-renewal and repair by turning senescent cells into NC progenitors via the SDF-1/CXCR4 pathway, which then triggers BMP activation to enhance cell growth and tissue repair. We deduce that HC-HA/PTX3-mediated rejuvenation may serve as a new medical therapy in the field of ophthalmology 137 (also reviewed in 138). We have indicated that HC-HA/PTX3 can inhibit α-SMA expression and nuclear pSMAD2/3 translocation in human corneal fibroblasts even with the addition of TGF-β1, and such inhibition is accompanied by a phenotypic reversal to younger keratocytes via the activation of BMP signaling 139. Further studies suggest that such phenotypic reversal is caused by cell aggregation initiated through SDF-1/CXCR4 signaling, showing CXCR4 nuclear translocation and upregulation of CXCR4 mRNA and protein, which activates canonical BMP signaling 139.

This new approach of epithelial cell growth in spheres mixed with NCPs helps illustrate how limbal progenitors renew and select their fate inside the limbal niche environment 140. Chemokine SDF-1 is part of a large family of small, secreted proteins that mediate white blood cell movement in immune defense 141. CXCR4 and CXCR7, identified as G-protein-coupled receptors (GPCRs), are the primary receptors for SDF-1 signaling 142, 143. However, CXCR4, but not CXCR7, regulates GPCR signaling 143. Our previous report indicates that limbal stromal cells express abundant CXCR4 but not CXCR7. CXCR4 attenuation by the CXCR4 inhibitor AMD3100 interfered with their reunion and generated separate, smaller spheres, which displayed significantly increased corneal differentiation and a notable decrease in holoclones 7. Moreover, IGF-1 modulates CXCR-4 in human lung microvascular endothelial cells, indicating that IGF-1 may be upstream of CXCR-4 144. SDF-1 may enhance HGF expression in MSCs, suggesting that HGF may be downstream of SDF-1 signaling 145. FGF2 may inhibit SDF-1 post-transcriptionally in bone marrow stromal cells via FGFR1 IIIc, suggesting that FGF2 is upstream of SDF-1 146. SDF-1 is a JUN-mediated factor critical in epithelial and mesenchymal interactions by mediating the expression of colony-stimulating factor 2 (CSF2) and fibroblast growth factor 7 (FGF-7) 147.

### Notch Signaling

During eye development, activation of Notch signaling has been shown to induce Pax6 and other eye-related genes in *Xenopus*. Postnatally, the Notch pathway is identified as an important regulator of the corneal transient amplified corneal epithelium (TACs) in fate decision 148, differentiation 149, and wound healing 150, 151. Notch1^-/-^ mice cause the corneal epithelium to develop into a hyperproliferative, skin-like epidermis 148. Overexpression of NICD in corneal epithelium-specific K14 transgenic mice promoted corneal epithelial wound healing 150. Interestingly, the Hes1-null mouse cornea showed a marked reduction in corneal thickness in stromal regions, with a single-cell layer of epithelial cells 152. This suggests that Hes1-mediated signaling may also affect corneal mesenchymal cells. Nevertheless, it is unclear whether the function of Hes1 in stromal cells could affect corneal epithelial SC function. Although Notch1/2 are mainly present at the corneal suprabasal epithelium but not at the limbal basal layer (epithelium) 149, 153, other groups have reported the opposite finding, in which membrane Notch1 was found in the limbal basal and subjacent suprabasal epithelium 154. *In vitro*, Notch inhibition by DAPT decreases differentiation in limbal epithelium alone, as demonstrated by decreased expression of K12+ with an increase of p63α+ cells and cell proliferation in rats 155 and humans. Notch inhibition does not prevent the limbal basal epithelium from stratifying and differentiating in a human cornea air-lifting model. Interestingly, the reunion of rLNC and rLEPC on a 3D Matrigel model can reduce nuclear Notch-NICD to prevent SC epithelium from K12 differentiation 155. However, none of the studies have described whether multiple Notch signaling events occur between LEPCs and LNCs, as well as within LNCs and LEPs, to regulate the stepwise transition of limbal basal SCs located deep within the crypt into TACs during corneal fate determination. Also, it remains unclear what role Notch signaling plays between LNCs in vasculogenesis.

Our findings in cross-sectioned human corneoscleral rims demonstrated (1) that membrane Notch receptors are predominantly present in the corneal as well as conjunctival epithelium but absent from the limbal basal epithelium. Our data further suggest that (2) NICD staining is predominantly localized to the nuclei of the suprabasal layers of the corneal and conjunctival epithelium, is weakly expressed in the limbal suprabasal epithelium, and is absent from the limbal basal epithelium, indicating that NICD-Notch signaling is suppressed in the limbal basal epithelial layer. Furthermore, we further found that (3) Notch3 and Jagged1 are constitutively expressed in the limbal basal epithelium and its subjacent mesenchymal cells. Consistently, (4) collagenase-isolated limbal clusters revealed weak nuclear NICD expression in PCK+ cells. Interestingly, the PCK-negative population (non-epithelial) contains a mixture of nuclear NICD(+) cells (white arrows) and NICD(-) cells. Our preliminary data collectively suggest that Notch3/Jagged1 may play a different role from Notch1 in the limbal epithelium and mesenchymal cells. Recently, we have reported that P10 LNCs on HC-HA/PTX3 induce cell aggregation as well as nuclear Pax6 expression with NC phenotypes and potential 137.

Expression of Hes1 has been demonstrated to be mediated through Notch-dependent and Notch-independent pathways to promote angiogenesis and neurogenesis. Oscillating Hes1 has been reported to be Notch-independent and regulated by BMP and LIF signaling in ES cells; by FGF2-JNK signaling in ES-derived neural progenitors 156; by NGF-NF-κB signaling with sustained Hes1 expression to maintain dendritogenesis 157; by VEGF-FLK-1-ERK (extracellular signal-regulated kinases) signaling, which promotes retinal progenitor proliferation and retinal ganglion cell fate specification 158; and by acetylation of Pax3 binding to the Hes1 promoter, which enhances neural SC maintenance 159. Hes1 has been known to regulate the undifferentiated status and maintenance of neural stem cell progenitors to promote proper neuronal differentiation and cell-cell-interactive lateral inhibition 160, 161. Hes1 expression, often in an oscillatory manner occurring approximately every two hours, has been demonstrated in fibroblasts and neural progenitors 162, 163. Without Hes genes, progenitor cells become specific neurons too early and too fast; as a result, the cells may not multiply fast enough to generate critical neurons and other supporting cells 164. Our data show that transient nuclear translocation of Hes1 occurs within 5 minutes of treatment with HC-HA/PTX3. Sustained expression of Hes1 enhances repression of pro-neuronal genes and maintains a low proliferative or quiescent state of cells 161. Notch-Hes1 signaling is responsible for the activation of HIF1α signaling and the phosphorylation of STAT3 at Tyr416 165. The mechanistic event responsible for the nuclear translocation of Hes1 remains unclear but may involve post-transcriptional regulation.

### Pax6 Signaling

The human Pax6 expression pattern has been well described 166. We have discovered that the gain of Pax6 function, a conserved transcription factor necessary for eye development, by late-passage LNCs may promote LNCs into NC progenitors 167. By an *in vitro* reunion assay, Pax6-overexpressing LNCs may prohibit differentiation of the corneal epithelium and promote the formation of a holoclone by LEPCs 167. Thus, we have concluded that nuclear Pax6 generated from LNCs has a major developmental impact on the maintenance of stromal and epithelial progenitor status in the limbal niche 167. In addition, we have noted that HC-HA/PTX3 may revert aging LNCs into functional stromal cells to support the generation of younger nuclear Pax6+ LEPs 137. Such reversal is closely associated with early-stage cell aggregation caused by the induction CXCR4 signaling, which then activates BMP signaling 137. We have also noted that CXCR4, rather than BMP signaling, dictates the reversal of aging nuclear Pax6- epithelial cells to younger nuclear Pax6+ epithelial cells 137. Thus, we conclude that HC-HA/PTX3 plays a critical role in the expansion of human LEPs, which may serve as a regenerative therapy for conditions such as LSCD 137 (also reviewed in 168).

### VEGF Signaling

Previously, Chiriac *et al*. (2010) reported that the induction pluripotent stem cells in heart tissue occurs through the activation of SDF-1, then vascular endothelial growth factor (VEGF), and then BMP2 signaling 169. Ping (2011) demonstrated that CD133+ cells have relatively higher CXCR4 mRNA and protein levels and have a greater capacity for migration when CXCR4 receptors are activated by the SDF-1 ligand than CD133- cells 170. Interestingly, in CD133+ glioma stem cells (GSCs), SDF-1 may trigger the PI3K and AKT pathways, which may then induce VEGF signaling. Blocking CXCR4 with AMD3100 decreases tumor growth and inhibits new blood vessel formation in mice implanted with brain cancer cells through inhibiting VEGF signaling. The action of VEGF is mediated though its receptor, VEGFR1. The receptor is regulated by oxidative stress, probably through the thioredoxin reductase/thioredoxin system in myeloid cells. In addition, thioredoxin-1 may promote macrophages from an M1 to an M2 phenotype (anti-inflammatory) and inhibit atherosclerosis (plaque buildup in arteries) 171. Furthermore, thioredoxin, a small oxidoreductase with an active dithiol-disulfide motif, may induce a wide range of essential biological processes, including the aryl hydrocarbon receptor (AHR), a ligand-activated protein that mediates gene activity. When activated, AHR may bind to target DNA to regulate gene transcription and translation, and it may also affect other transcription factors, such as AP-1, TFIIIC, and NF-κB, to regulate gene expression 172. In addition, thioredoxin-interacting protein (TXNIP) may inhibit thioredoxin via direct binding, serving as an important negative regulator of cellular redox homeostasis 173. Furthermore, cell-cell interactions regulate VEGF secretion via ERK/AKT signaling, and such activation is vital for activating paracrine signaling and promoting MSC growth 174. Interestingly, previous data show that the TGF-β1 may induce destabilization of cellular adhesion, a calcium-mediated biological process whereby epithelial cells form stable tissues and maintain their structure through E-cadherin regulation 175, and that osteoclasts may activate TGF-β from its inactive form in matrix-bound latent complexes in bone resorption 176.

Moreover, it is unclear whether E-cadherin is involved in VEGF regulation and aggregation induced by immobilized HC-HA. Similarly, it is unknown whether other similar junctional molecules, for example, N-cadherin and Cx43, also mediate aggregation induced by immobilized HC-HA. It has yet to be determined whether TGF-β is involved in aggregation induced by immobilized HC-HA and, if so, whether such an action occurs through destabilization of E-cadherin. Since the aggregation is a prerequisite for bone formation, it is not known whether activation of TGF-β signaling by immobilized HC-HA is also involved in controlling bone formation and, if so, whether such an activation of TGF-β signaling is involved in SOX9-mediated chondrocyte differentiation.

### VCAM-1 and ICAM-1 Signaling

The activation of EPCs from the bone marrow is also regulated by a variety of specific signals, including interleukins 177, 178, α4β1 integrin and its binding partner, vascular cell adhesion molecule-1 (VCAM-1), as well as its regulator, integrin-linked kinase (ILK), which regulates postnatal blood vessel formation via driving endothelial progenitors to oxygen-depleted tissues 179. IL-8 and MCP-1 may mediate cell movement in the case of developing clogged arteries 180. Activation of LFA-1 as well as VLA-4 converts interactions with ICAM-1 and VCAM-1 into firm adhesion 181.

### Other Signaling

Calderon *et al*. (2012) established a complex model to grow artificial T-cell-producing (thymopoietic) tissue compartments inside mouse embryos 182. In this article, the authors suggest that the thymic epithelium does not have blood-producing function but is accessible to blood progenitors, a process mediated through chemokines such as Ccl25 and SDF-1α. Their findings are important because this modular system resembles the limbus, where SDF-1α is upregulated by LEPs. Previously, it was reported that YAP maintains limbal niche stemness 183. Inhibition of YAP is considered a treatment mechanism for a burned corneal surface, using collagenase to restore the mechanical properties of the tissue and to support LEPs 184. Using a Sox10-mutant mouse model and co-culture experiments *in vitro*, the authors demonstrated that Sox10 in LNCs promotes KIT ligand expression, which then activates KIT-AKT signaling to inhibit apoptosis by reducing the activity of caspase 3. The findings indicate that the KIT-AKT pathway mediated by Sox10 is important in limbal niche maintenance and the survival of limbal epithelial stem cells (LESCs), providing an understanding of limbal niche roles and subsequent therapeutic approaches for LSCD 168, 185-193. Other evidence indicates that Wnt proteins are specific cellular signaling molecules that mediate how embryos may grow, how cells may proliferate, and how certain cells become specific tissues 194, 195. Canonical Wnt may decrease the production of receptor activator of NF-κB ligand protein, which may slow down bone breakdown 196. Wnt activity may support self-renewal and maintenance of blood stem cells 197. In addition, MMPs are crucial in the homing process, which involves the SDF-1/CXCR4 axis. We have noted that the expression of MMP-1 and MMP-3 is downregulated via HC-HA in cultivated CCh stromal cells.

The major signal pathways for LN reprogramming is summarized in Table 1.

### Future Directions

Future directions in limbal niche research focus on reconstructing the niche using bioengineered scaffolds, MSCs, alternative cell sources (such as hair follicle- and dental tissue-derived cells), and signaling factors; improving transplantation strategies through advanced single-cell technologies; and exploring novel therapies, including angiogenesis modulation and cellular reprogramming, to prevent corneal disease. Key goals are to restore stem cell function, control inflammation, and develop more effective, less invasive treatments for LSCD.

Key Research Areas:

1. Niche Reconstruction and Bioengineering: a) Mimicking the Niche: To develop artificial ECM or scaffolds that resemble the limbal niche's topography and signals (such as Wnt/BMP) to promote stem cell growth. b) Native Niche Cell Co-Culture: Reconstituting the limbal niche *in vitro* by co-culturing LESCs with stromal niche cells to understand self-renewal and quiescence.

2. Advanced Cell Therapies: a) MSCs: Using MSCs (from bone marrow, dental pulp, etc.) to modulate inflammation, scarring, and angiogenesis, potentially in co-transplantation with LESCs. b) Alternative Sources: Exploring hair follicles, teeth (dental pulp), and skin as sources of multipotent cells to generate corneal epithelial cells. c) Reprogramming: Reprogramming existing adult corneal cells or pluripotent stem cells into LSC-like cells using gene editing and specific developmental stimuli.

3. Molecular Signaling: a) Single-Cell Technology: Using single-nucleus or single-cell transcriptomics to monitor molecular identity within the human limbal niche and characterize novel markers. b) Signaling Pathways: Investigating how Wnt, BMP, and other signaling pathways regulate LESC self-renewal and fate decisions, as well as the role of persistent inflammation.

4. Therapeutic Factor-Based Approaches: a) Soluble Factors: Delivering specific growth factors as well as cytokines (and extracellular vesicles, such as exosomes) from niche cells to stimulate corneal regeneration. b) Angiogenesis Modulation: Targeting pathological neovascularization, as the limbus normally acts as a barrier to blood vessel invasion.

5. Improving Surgical Techniques: Developing faster, more efficient surgical methods (e.g., simplified mucosal transplantation) and improved scaffolds (such as the amniotic membrane) for cultivated cells.

Overarching Goals: a) Move beyond simple cell transplantation to restore the *entire* niche microenvironment. b) Reduce reliance on donor corneas and invasive procedures. c) Address inflammation and maintain immune privilege to ensure long-term success.

## Conclusion

LNCs are major stromal progenitors in the limbal microenvironment, exhibiting adipogenic, chondrogenic, and osteogenic potential. LNCs express ESC markers, for example, Sox2, Nanog, and Oct4, and mesenchymal progenitor markers, for example, CD90. In addition, LNCs interact with LEPs to support their aggregation and differentiation. Furthermore, the HC-HA/PTX3 complex may maintain LNC quiescence and stemness as well as support niche-derived progenitor cells (NCPCs). Key signaling pathways that maintain limbal progenitor status include BMP-mediated MET, SDF-1/CXCR4-driven cell aggregation and holoclone formation, Notch signaling-mediated fate determination, differentiation, and wound healing, Pax6-mediated maintenance of the NCPC state, and Rho GTPase (Rac/RhoA)-governed cytoskeletal dynamics.

Therefore, understanding the regeneration of adult limbal progenitors and the related mechanisms may pave the way for cell-based therapies of various eye diseases caused by stem cell deficiency or malfunction.

## Figures and Tables

**Figure 1:**
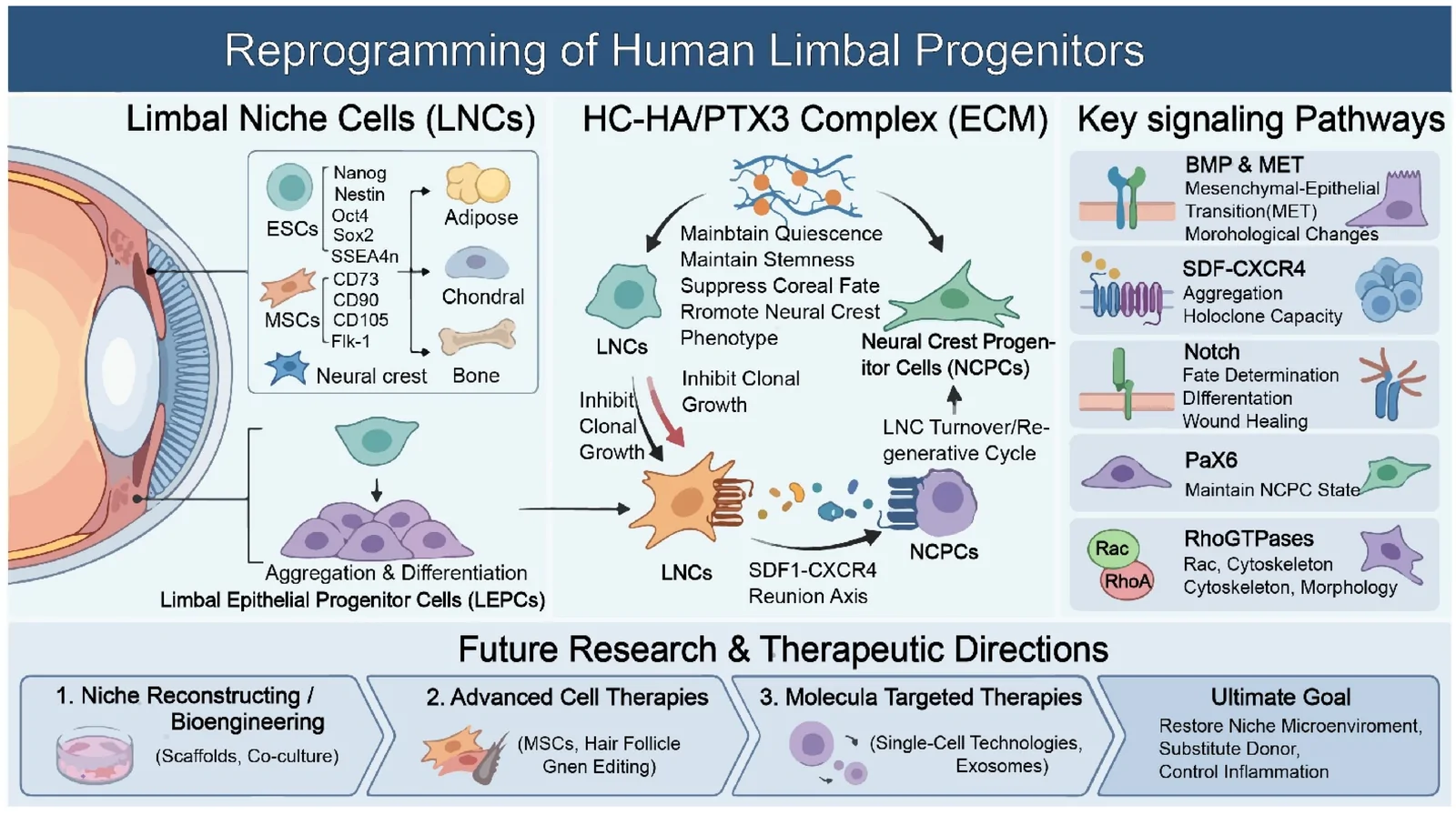
Overview of limbal niche progenitor reprogramming and key regulatory mechanisms. This diagram illustrates the key components and regulatory mechanisms governing limbal niche homeostasis and progenitor reprogramming. (Left) Limbal niche cells (LNCs) and limbal epithelial progenitors (LEPs) reside in the limbal microenvironment. LNCs exhibit multi-lineage differentiation potential (adipogenic, chondrogenic, and osteogenic) and express epithelial stem cell markers (Nanog, Oct4, and Sox2) and stromal progenitor markers (CD73, CD90, CD105, Flk-1), with the capacity to form neural crest-like cells. LNCs interact with LEPs to support their aggregation and differentiation. (Middle) The HC-HA/PTX3 complex (a native extracellular matrix component) maintains LNC quiescence and stemness, suppresses corneal fate commitment, and promotes a neural crest phenotype, thereby supporting the maintenance of niche-derived progenitor cells (NCPCs). Conversely, dysregulated LNCs inhibit clonal growth, whereas the SDF-1/CXCR4 axis facilitates LNC turnover and the regenerative cycle toward NCPCs. (Right) Key signaling pathways regulating limbal progenitors are summarized, including BMP-mediated mesenchymal-epithelial transition (MET) and morphological changes, SDF-1/CXCR4-driven cell aggregation and holoclone formation, Notch signaling for fate determination, differentiation, and wound healing, Pax6 for maintaining the NCPC state, and Rho GTPases (Rac/RhoA) governing cytoskeletal dynamics and morphology. (Bottom) Future research and therapeutic directions include (1) niche reconstruction and bioengineering (scaffolds, co-culture systems), (2) advanced cell therapies (using mesenchymal stem cells, hair follicle cells, and gene editing), and (3) molecularly targeted therapies (single-cell technologies, exosomes), to achieve the ultimate aim of repairing the damaged limbal niche, reducing donor dependency, and controlling inflammation in limbal stem cell deficiency.

**Table 1:** Summary of Signaling Pathways for hLNCs Reprogramming

Signaling Pathway	Key Molecules	Brief Biological Functions
BMP signaling	BMP, pSmad1/5/8, Bmpr1a-Acvr1	Regulates mesenchymal-to-epithelial transition (MET); maintains cell quiescence; coordinates with the Wnt pathway to mediate limbal epithelial progenitors (LEPs) growth in colonies; mediates the stemness-maintaining effect of HC-HA/PTX3.
Wnt signaling	Canonical Wnt, noncanonical Wnt	Modulates the fate determination of LEPs; canonical Wnt promotes clonal growth of LEPs; noncanonical Wnt is activated by HC-HA/PTX3 to inhibit corneal differentiation.
SDF-1/CXCR4 signaling	SDF-1, CXCR4, CXCR7	Mediates cell aggregation and sphere formation between limbal niche cells (LNCs) and LEPs; triggers nuclear translocation of CXCR4; acts as an upstream regulator of the BMP pathway; prevents corneal epithelial differentiation.
Notch signaling	Notch1/2/3, Jagged1, NICD, Hes1	Regulates corneal epithelial regeneration and damaged tissue repair; inhibits LEP differentiation; maintains limbal niche homeostasis; Hes1 participates in cell quiescence and angiogenesis regulation.
Pax6 signaling	Pax6	Promotes neural crest progenitor characteristics in senescent LNCs; suppresses corneal epithelial differentiation; maintains the self-renewal capacity of LEPs and the stemness of the limbal niche.
Rho GTPases signaling	RhoA, Rac1, Cdc42, ROCK, MLC2	Regulates cytoskeletal rearrangement and cell morphological changes; participates in MET and cell contraction; controls cell motility and cell-cell interactions within the limbal niche.
VEGF signaling	VEGF, VEGFR1, Flk-1	Modulates angiogenesis; interacts with the SDF-1/CXCR4 axis to regulate the paracrine activity of stromal cells; participates in tissue repair and vascular niche construction.
VCAM-1/ICAM-1 signaling	VCAM-1, ICAM-1, VLA-4	Mediates cell adhesion and trafficking; participates in endothelial progenitor cell mobilization and vascular microenvironment remodeling in limbal tissue.
